# Discovery of biological markers for schizophrenia based on metabolomics: a systematic review

**DOI:** 10.3389/fpsyt.2025.1540260

**Published:** 2025-03-28

**Authors:** Gaolei Yao, Jingchun Zeng, Yuan Huang, Huipeng Lu, Junjiao Ping, Jing Wan, Tingyun Jiang, Fuyuan Deng, Chenyun Li, Xinxia Liu, Chunzhi Tang, Liming Lu

**Affiliations:** ^1^ Clinical Research and Big Data Laboratory, South China Research Center for Acupuncture and Moxibustion, Medical College of Acu-Moxi and Rehabilitation, Guangzhou University of Chinese Medicine, Guangzhou, China; ^2^ Rehabilitation Centre, The First Affiliated Hospital of Guangzhou University of Chinese Medicine, Guangzhou, China; ^3^ Rehabilitation Centre, Guangdong Clinical Research Academy of Chinese Medicine, Guangzhou, China; ^4^ Department of Acupuncture, Shaoguan Hospital of Traditional Chinese Medicine, Shaoguan, China; ^5^ Department of Psychiatry and the Research Laboratory, The Third People’s Hospital of Zhongshan, Zhongshan, China

**Keywords:** schizophrenia, metabolomics, metabolite, pathway enrichment, systematic review

## Abstract

**Introduction and methods:**

To discover biomarkers for schizophrenia (SCZ) at the metabolomics level, we registered this systematic review (CRD42024572133 (https://www.crd.york.ac.uk/PROSPERO/home)) including 56 qualified articles, and we identified the characteristics of metabolites, metabolite combinations, and metabolic pathways associated with SCZ.

**Results:**

Our findings showed that decreased arachidonic acid, arginine, and aspartate levels, and the increased levels of glucose 6-phosphate and glycylglycine were associated with the onset of SCZ. Metabolites such as carnitine and methionine sulfoxide not only helped to identify SCZ in Miao patients, but also were different between Miao patients and Han patients. The decrease in benzoic acid and betaine and the increase in creatine were the notable metabolic characteristics of first-episode schizophrenia (FESCZ). The metabolite combination formed by metabolites such as methylamine, dimethylamine and other metabolites had the best diagnostic effect. Arginine and proline metabolism and arginine biosynthesis had a clear advantage in identifying SCZ and acute SCZ. Butanoate metabolism played an important role in identifying SCZ, toxoplasma infection and SCZ comorbidity. Biosynthesis of unsaturated fatty acids was also significantly enriched in the diagnosis and treatment of SCZ.

**Discussion:**

This study summarizes the current progress in clinical metabolomic research related to SCZ, deepens understanding of the pathogenesis of SCZ, and lays a foundation for subsequent research on SCZ-related metabolites.

**Systematic review registration:**

https://www.crd.york.ac.uk/PROSPERO/home, identifier CRD42024572133.

## 1 Introduction

Schizophrenia (SCZ) is a clinically common severe mental illness, affecting approximately 1 in every 300 people, accounting for 1% of the global population. It is one of the top 10 leading causes of disability worldwide (1). Studies show that men are more likely to suffer from SCZ at a younger age (2). Symptoms of SCZ begin to appear between the ages of 20 and 29 and include delusions, hallucinations, and a lack of coordination between thinking and behavior (positive symptoms), and insociability, world-weariness, anorexia, and decreased energy (negative symptoms), and decreased attention and memory (cognitive dysfunction) (3). The social misunderstanding and stigmatization of patients with SCZ further aggravate their psychological burden and limit their social participation and rehabilitation opportunities. There is evidence that the life expectancy of people with SCZ is reduced by 15 to 25 years due to suicide, accidents, antipsychotic medications, lower quality of life, and various comorbidities such as cardiovascular disease, hypertension, and diabetes, inadequate medical care, and premature aging (4).

As of this writing, the diagnosis of SCZ remains overly subjective, not only because the symptom spectrum is complex and similar to other mental disorders, but also because of a lack of objective disease biomarkers (5). Moreover, the experience of clinicians is uneven, and it is difficult to make an accurate diagnosis based solely on a subjective understanding of symptoms. Thus, there is a significant clinical demand for biomarkers that can aid in the diagnosis of SCZ (6). The lack of reliable biomarkers leads to delays in diagnosis, preventing patients from receiving timely and effective treatment. Therefore, identifying biomarkers associated with SCZ is crucial for early diagnosis and treatment.

Metabolomics is an important part of system biology, and its research objects are small molecules with molecular weight less than 1,000 Da, such as sugars, organic acids, lipids, amino acids and aromatic hydrocarbons (7). Metabolomics techniques have been widely applied in basic research in recent years, such as nuclear magnetic resonance (NMR) technology, gas chromatography-mass spectrometry (GC-MS), liquid chromatography-mass spectrometry (LC-MS), and capillary electrophoresis-mass spectrometry (CE-MS) (8). Principal component analysis and partial least squares discriminant analysis provide a statistical foundation for identifying differential metabolites. According to varying research objectives, metabolomics can be classified as either non-targeted metabolomics or targeted metabolomics (9). In non-targeted metabolomics, the entire metabolome of an organism is comprehensively searched to detect any metabolic characteristics in which there are significant changes between the experimental and control groups; while in targeted metabolomics, target metabolites are studied to verify biomarkers. In metabolomics, there is a terminal effect and an amplification effect, which can reflect organisms’ disease physiological states more directly and sensitively than genomics or transcriptomics. Compared with genomics and proteomics, metabolomics studies have fewer substances and a simpler information bases, which gives metabolomics a unique advantage in disease diagnosis and biomarker discovery. The use of metabolomics to study SCZ has attracted more and more attention in recent years, and related research is on the rise. However, the quality of these studies has been mixed, and the aims and methods of these studies have been multifarious, so it is currently necessary to consider many studies together to form consensus.

To this end, we used a systematic review to summarize current research progress in SCZ at the metabolomics level, elaborate on the metabolite changes related to SCZ, reveal the pathogenesis of SCZ, and determine a metabolite combination that can identify patients with SCZ, so as to lay a foundation for a more accurate diagnosis of SCZ. Moreover, this study also explores the current challenges in metabolomics research related to SCZ, providing direction for future studies. Through this systematic review, insight and ideas for the diagnosis, prognosis, and disease detection of SCZ are provided.

## 2 Methods

This systematic review was registered with PROSPERO (CRD42024572133) in accordance with the requirements of a routine systematic review.

### 2.1 Literature search

We searched for metabolite studies related to SCZ in PubMed, Embase, and Web of Science databases, with a literature time span from database establishment to August 2024. The search strategy was formulated using a combination of subject words and free words. The main subject terms were: “schizophrenias”, “dementia praecox”, “schizophrenic disorders”, “disorder,schizophrenic”, “disorders, schizophrenic”, “schizophrenic disorder”, “metabolomics”, “metabolome”, “metabolic flux analysis”, “metabolic profiling”, “metabolic signature”, “metabolic biomarker” and “meta-bolic profile”. The literature was searched separately by two researchers, and if there was any disagreement, it was decided by senior literature experts. The specific search strategy can be found in the **Supplementary Materials**. We searched for relevant literature based on the pre-established search strategy, and then imported the retrieved literature into NoteExpress (version 4.1.0).

### 2.2 Inclusion and exclusion criteria

#### 2.2.1 Inclusion criteria

1. Study subjects: patients with SCZ diagnosed by the Diagnostic and Statistical Manual of Mental Disorders (DSM) or International Classification of Diseases (ICD)-10.
2. Research types: case-control, randomized controlled trials, etc.
3. Research content: the diagnosis and prognosis of SCZ through metabolomics.
4. Data types: human-involved clinical studies, including clinical and metabolomic data.
5. Language type: English.

#### 2.2.2 Exclusion criteria

Duplicate literature or literature published with already-published data; literature involving other diseases or literature not involving metabolite studies; comments and letters; animal experiment literature; review, Mendelian randomization research, protocol, and case reports; literature with incomplete data or data that cannot be extracted; research on intervention mechanisms; literature that was unavailable in full-text format.

### 2.3 Information extraction

After determining the final included literature based on the inclusion and exclusion criteria, we began to extract relevant information from the included studies. First, we recorded the extracted author name, publication year, journal, country, research subjects, gender, group settings, research type, sample size, analysis platform, research purpose, sample type, whether it was a targeted study, any changes in related metabolites, and the pathways involved in Excel. If a study included both case-control studies and self-controlled studies before and after intervention, it was divided into two studies to extract the data.

### 2.4 Methodological quality assessment

We used QUADOMICS (Quality Assessment of Diagnostic Accuracy Studies for Omics), a quality assessment tool specifically modified for omics studies, in assessing the methodological quality of studies related to differential metabolites that can be used in the diagnosis of SCZ (10). This tool evaluates the methodological quality of the literature from 16 dimensions: patient selection, selection of diagnostic criteria, randomization methods, description of specimen types, interpretation of results, reproducibility of trial protocols, and rigor of trial execution. As for studies related to metabolites associated with SCZ prognosis, we used the QUIPS (Quality In Prognosis Studies tool for evaluation (11). This tool assesses the quality of a study from six perspectives: selection of participants, study attrition, measurement of diagnostic factors, measurement of outcomes confounding factors of the study, and statistical analysis and reporting. Documents that meet 75% and above of the items are considered high-quality documents, those that meet 50-75% are considered medium-quality documents, and those that meet less than 50% are considered low-quality documents (12).

### 2.5 Statistical analysis

In order to comprehensively summarize the current progress of metabolomics research in SCZ, we recorded the countries, specimen types, metabolite detection technologies, related metabolites and metabolic pathways involved in the study, and used descriptive statistics to analyze their frequencies and percentages. Then, we used visualization to display them.

We conducted a summary statistical analysis of the metabolites involved in high-quality literature, collated information on the accuracy (Area Under Curve, AUC) of metabolite combinations in the diagnosis and prognosis analysis of SCZ and completed the metabolic pathway enrichment using MetaboAnalyst 6.0 online software (http://www.metaboanalyst.ca/). This comprehensive analysis of significantly enriched pathways in high-quality literature revealed the pathological mechanisms associated with SCZ.

We used Adobe Illustrator (2024 version) to create a flowchart for literature search and screening, and mapped the study sites via Wei Sheng Xin (https://www.bioinformatics.com.cn/). Other plots were completed in Rstudio (version 4.4.2); the histogram of specimen classification and the enrichment map of metabolite pathways for the high-quality literature were conducted in the ggplot2 package (version 3.5.1). We drew the literature quality assessment chart with the dplyr package (version 1.1.4) and ggplot2 package (version 3.5.1) and prepared the high-quality literature accumulation histograms using the reshape2 package (version 1.4.4) and ggplot2 package (version 3.5.1).

## 3 Results

### 3.1 Literature search and screening results

**Figure 1:**
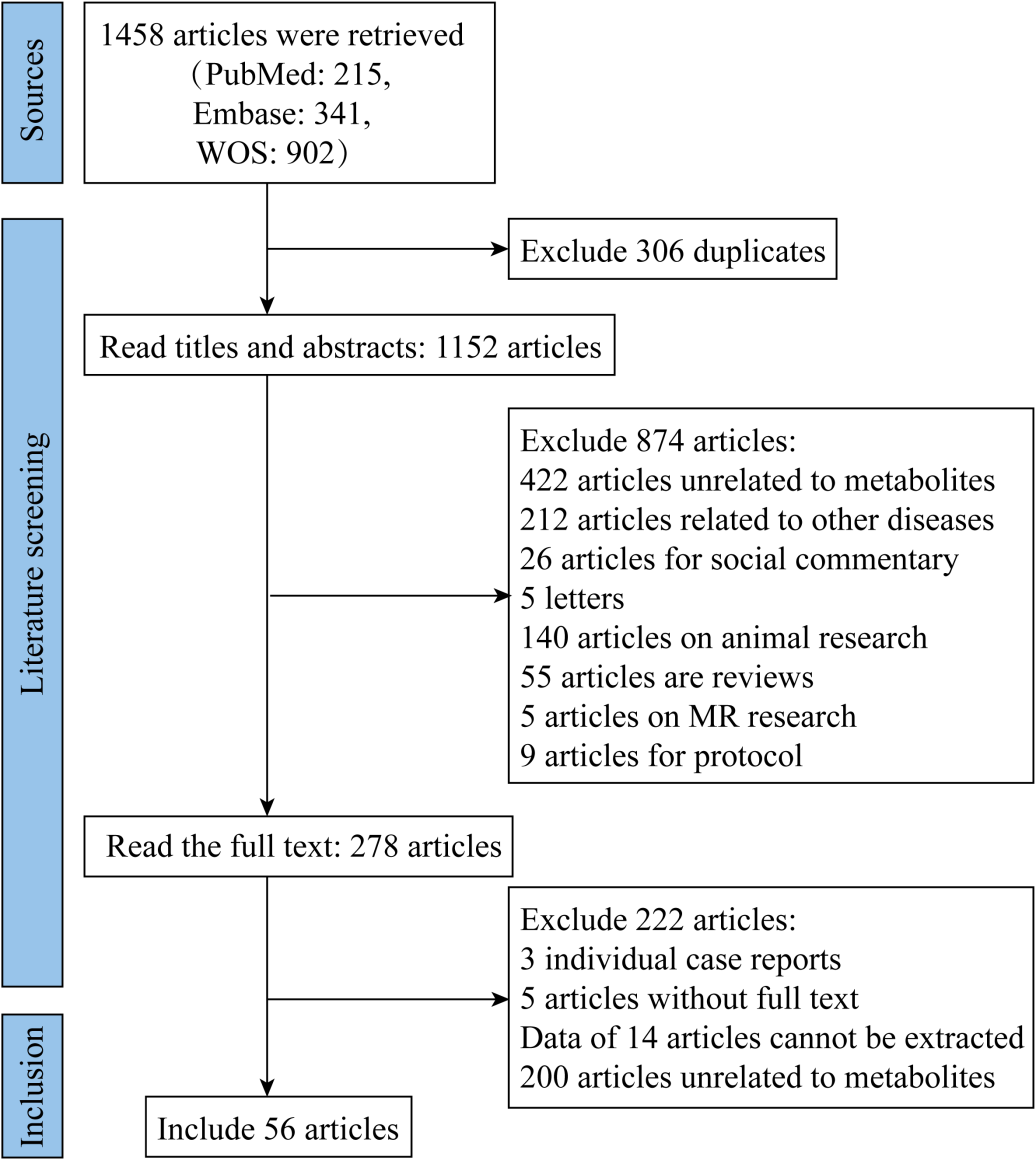
Flowchart of literature retrieval and screening.

Based on the pre-determined search strategy, we retrieved 215, 341, and 902 articles from the PubMed, Embase, and Web of Science databases, respectively. After importing the above 1,458 documents into NoteExpress, 306 duplicate documents were eliminated by author, publication year and title. After reading the title, abstract and full text to eliminate unqualified documents, 56 documents were ultimately included. The literature search and screening process is depicted in **Figure 1**.

### 3.2 Basic characteristics of the included literature

**Figure 2:**
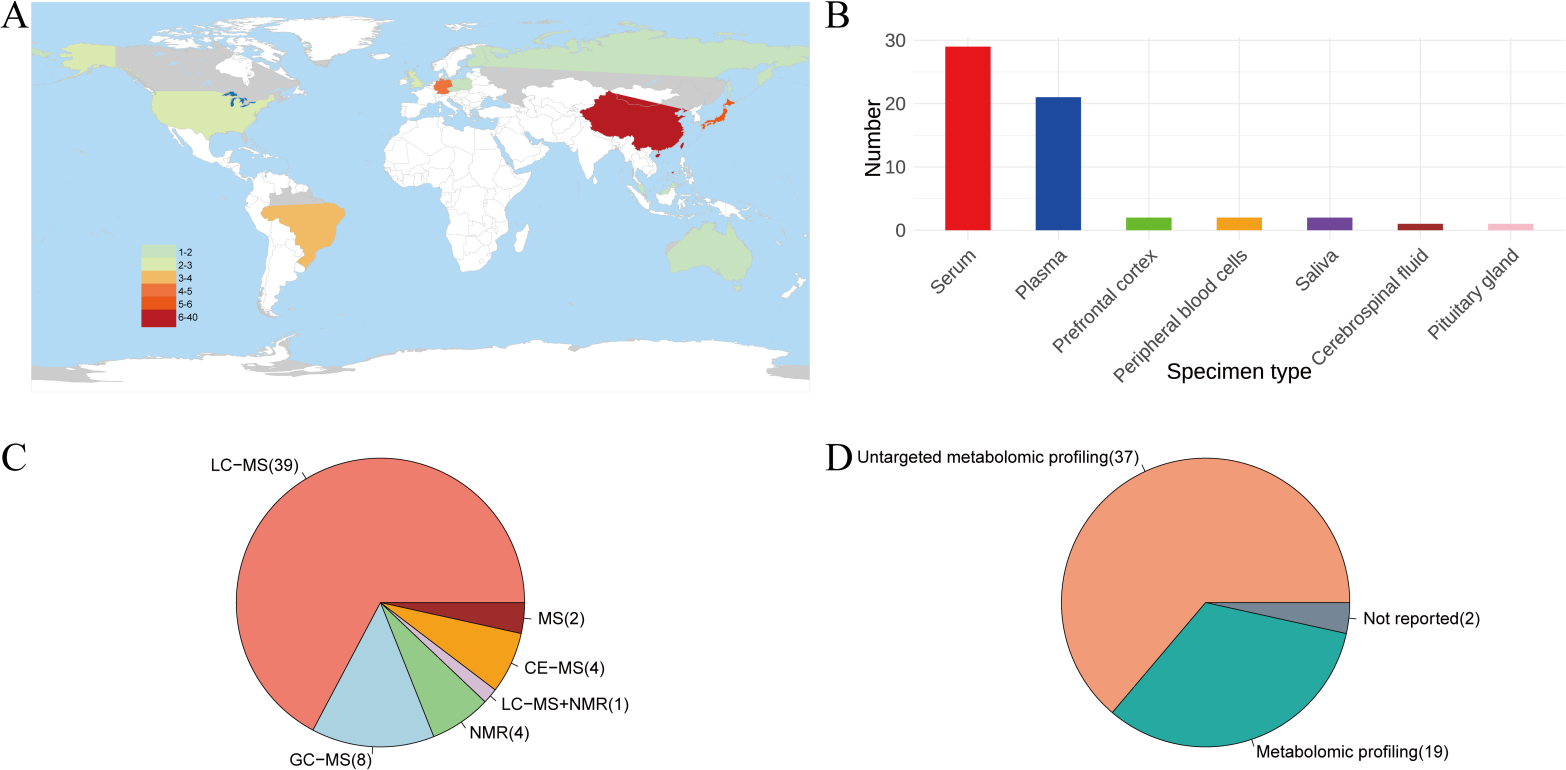
Results of the basic characteristics of the included literature. **(A)**. Distribution of research locations included in the literature. **(B)**. Classification of specimens involved in the study. **(C)**. Round pie chart of the metabolite detection technique. **(D)**. Round pie chart of whether to target detection. NMR, nuclear magnetic resonance; GC-MS, gas chromatography-mass spectrometry; LC-MS, liquid chromatography-mass spectrometry; CE-MS, capillary electrophoresis-mass spectrometry; MS, mass spectrometry.

The 56 articles included in this study involved 6,772 participants, with one study only including male patients and two studies only including female patients. Since two of the studies (13, 14) included both case-control and pre-and post-intervention self-control studies, each study was split into two studies for statistical analysis. The sample size for a single study ranged from 29 to 481. The 58 study sites were distributed in Japan (5), China (36), the United Kingdom (2), the United States (2), Brazil (3), Germany (4), and the remaining 6 were completed in Poland, Australia, the Czech Republic, Russia, Finland and Malaysia, respectively. 52 studies were designed to identify any metabolites that differed between people with SCZ and healthy controls. One study was conducted to compare the metabolites of SCZ between Chinese Han and Miao ethnic groups and the healthy population. Two studies involved first-episode schizophrenia (FESCZ). One study focused on patients with chronic schizophrenia (CSCZ). Three studies explored metabolite differences in untreated SCZ patients. What’s more, two studies explored the blood metabolites that differ in cognitively impaired and cognitively intact SCZ patients. One study explored the plasma differential metabolites of SCZ with violence tendency and SCZ without violence tendency. Another study investigated salivary metabolites in patients with clozapine-induced salivation and in patients without clozapine-induced salivation. Another explored the differential metabolites of the pituitary gland after autopsy in SCZ patients, and another explored metabolite differences in patients with SCZ caused by toxoplasma gondii infection. Two studies explored the differences in metabolites before and after intervention based on self-control before and after intervention. One study explored the metabolomic differences between SCZ with and without auditory hallucinations. 54 studies employed a case-control design, while two were cross-sectional studies. 51 studies were diagnostic, 5 were used for complication identification, and 2 involved prognostic analysis. Among the 58 metabolomics studies related to SCZ, 49 used blood as the specimen, which may be because blood sampling provides convenience for clinical research. In addition, 39 of these 58 studies performed metabolomics studies using LC-MS and 5 used NMR techniques to detect metabolites. In 37 studies, non-targeted metabolomics technology was used, and targeted technology was used in 19. Two studies did not report whether targeted technology was used. See **Supplementary Table S1**; **Figure 2** for details.

### 3.3 Quality evaluation results

**Figure 3:**
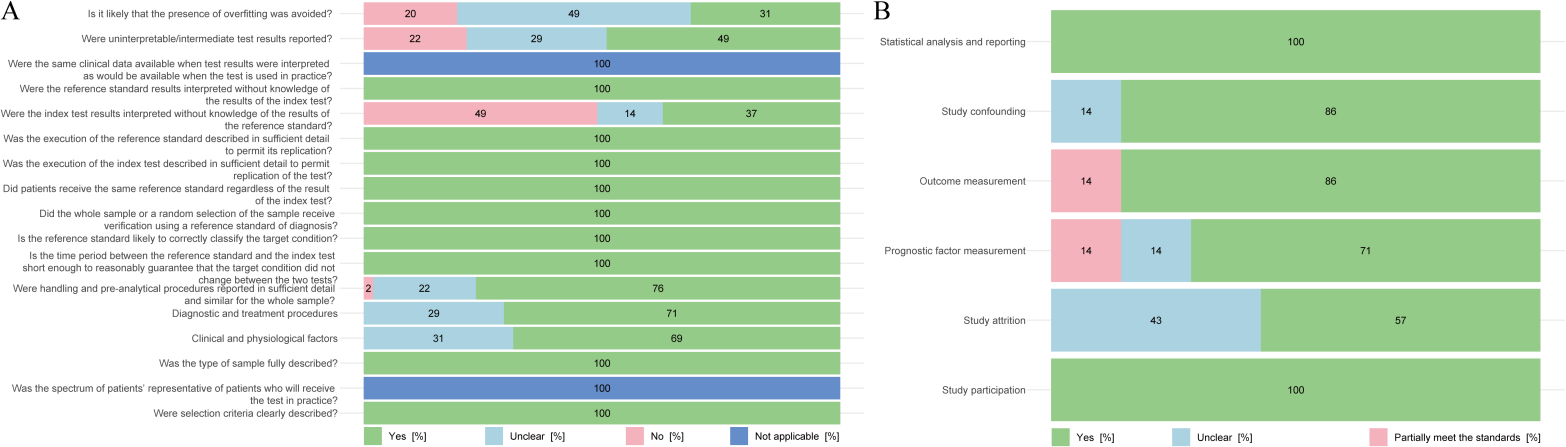
Quality assessment chart of included literature. **(A)** Results of a methodological quality assessment of studies related to differential metabolites that can be used for the diagnosis of schizophrenia. **(B)** Results of a methodological quality assessment of studies related to differential metabolites that can be used to predict the prognosis of schizophrenia.

Of the 51 diagnostic studies, 39 met the scoring criteria on at least 12 dimensions and were rated as high quality, while the remaining 12 were rated as medium quality. One of the two prognostic analyses adequately controlled for confounders and considered dropout to reduce bias. Of the 7 studies that identified complications and analyzed prognosis, 5 were high-quality and 2 were intermediate-quality. See **Figure 3** for specific information.

### 3.4 Overview of metabolic biomarkers associated with SCZ

One study (15) explored the metabolic differences in serum levels between patients with CSCZ and healthy individuals, and found that glutamate metabolism and the urea cycle were severely downregulated after onset. Meanwhile, another study (16) discovered significant differences in serum metabolites between Han and Miao schizophrenics and healthy controls in China. The metabolites Han and Miao patients had in common were: fatty acids and derivatives (e.g., indole-3-butyric acid, 2-oxovalic acid, eicosapentaenoic acid), amino acids (e.g., glutamate, pyroglutamic acid, proline, taurine), and other types (e.g., bilirubin, uric acid, α-tocopherol). The pathways with significant metabolic alteration in Miao patients were the arachidonic acid metabolism and α-linolenic and linoleic acid pathway. Meanwhile in Han Chinese patients, arginine and proline metabolism, arachidonic acid, alanine metabolism, the urea cycle, and glycine and serine pathways had changed significantly. Lipid and amino acid metabolism were common metabolic pathways across different ethnic groups of patients. Two studies (17, 18) compared the differences in plasma metabolites between patients with FESCZ and a normal population. They discovered that in patients with FESCZ, levels of creatine, isocitrate, succinic acid, itaconic acid, and L-2-hydroxyglutarate were all upregulated, while levels of betaine, nonanoic acid, benzoic acid, perillic acid, L-3,4-dihydroxyphenylalanine (L-dopa), dopamine 3-O-sulfate, norepinephrine sulfate, and normetanephrine were downregulated. Additionally, alterations were observed in the metabolic pathways of homocysteine metabolism, creatine kinase-emia, oxidative stress, aromatic amino acid metabolism, glutamate metabolism, nucleotide metabolism, and the tricarboxylic acid cycle. Two studies (19, 20) compared the metabolic differences between untreated SCZ patients and healthy individuals, finding that the differential metabolites were concentrated in aspartic acid, carnitine, lithocholic acid, lyso-phosphatidylcholine, lyso-phosphatidylethanolamines, phosphatidylcholine, phosphatidylethanolamine, and γ-aminobutyric acid (GABA). It was worth mentioning that 41 studies (6, 13, 21–59) reported a series of differential metabolites related to SCZ, including eicosanoids, docosahexaenoic acid, eicosapentaenoic acid, ethanamide, eicosadienoic acid, linolic acid, arginine, tryptophan and other metabolites. Two studies (60, 61) reported changes in metabolites induced by cognitive function, including sphinganine, D-glutamine, pyrrolidonecarboxylic acid, choline and creatine, lactic acid, aspartic acid, erythronic acid and 2-furoic acid. What’s more, one study (62) reported metabolites associated with violent tendencies in SCZ, including vanillylmandelic acid, glycerol, glyceraldehyde, malic acid and L-methionine. One clinical trial (63) explored the metabolite changes induced by salivation in patients with SCZ induced by clozapine such as guanosine, adrenaline, pyroglutamate, histidine, deoxycytidine, histamine, D-ornithine, isoleucyl-glutamate, hypoxanthine and kynurenine. A study from Germany (64) investigated SCZ from the perspective of pituitary metabolites and found differences in metabolites such as signal-induced proliferation-associated protein 1, protein KIAA1199, fibrinogen beta chain, prolactin, and secretagogin. Another study (65) revealed a correlation between toxoplasma infection and the onset of SCZ, involving metabolites such as alpha hydroxyglutaric acid, caprolactam, 3,30-thiopropionic acid, adenosine monophosphate, inosine, hypoxanthine and xanthine. A different study (66) analyzed the metabolites related to auditory hallucination, and found that metabolites such as phenylalanine, pyrroline hydroxyl carboxylic acid and pyruvate were related to auditory hallucination. Two studies (13, 14) reported metabolite changes in SCZ patients after antipsychotic treatment, including: tyrosine, linoleic acid, palmitic acid, oleic acid, tryptophan, uric acid, lactate, aspartate, glycine, myo-inositol, glucuronic acid, stearic acid, glycerol, lactobionic acid, lysoPC, sulfate, linoleic acid, oleic acid, palmitoleic acid, γ-linolenic acid, oxoglutaric acid, and androsterone.

### 3.5 Metabolites associated with SCZ in high-quality literature

**Figure 4:**
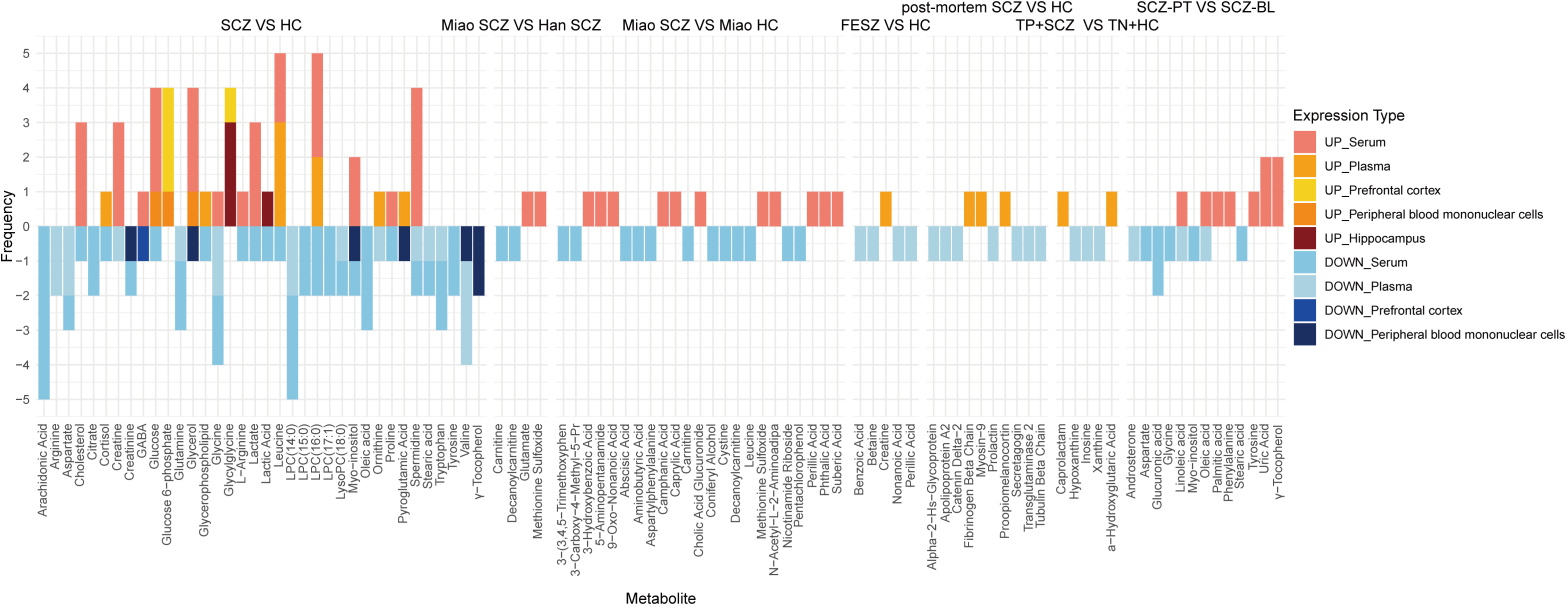
Accumulation histograms of metabolites associated with SCZ in high quality literature. Lyso-PC, Lyso-phosphatidylocholine; GABA, γ-aminobutyric acid.

Frequency analysis of the metabolites involved in high-quality research showed the commonalities of metabolomics research in different backgrounds (see **Figure 4**). Compared with the healthy control population, the main up-regulated metabolites of SCZ were glucose 6-phosphate and glycylglycine; the main down-regulated metabolites were arachidonic acid, arginine, aspartate, citrate, creatinine, glutamine, LPC (14:0), LPC (15:0), LPC (17:1), LysoPC (18:0), oleic acid, stearic acid and tryptophan. Metabolites with up-regulation in some studies and down-regulation in others included: cholesterol, cortisol, creatine, GABA, glucose, glycerol, L-arginine, lactic acid, lactate, leucine, LPC (16:0), myo-inositol, ornithine, proline, pyroglutamic acid. There were also differences in metabolites between Miao and Han SCZ patients. Glutamate and methionine sulfoxide were up-regulated more often in Miao SCZ patients than in Han SCZ patients, and carnitine and decanoylcarnitine were more often down-regulated in Miao SCZ patients. Within the Miao population, there were also differences in metabolites between SCZ patients and healthy people. Compared with the healthy Miao population, on the one hand, the main up-regulated metabolites in Miao SCZ patients were: phthalic acid, 3-hydroxybenzoic acid, methionine sulfoxide, N-acetyl-L-2-aminoadipa, suberic acid, 5-aminopentanamide, 9-oxo-nonanoic acid, caprylic acid, camphanic acid, perillic acid and cholic acid glucuronide, on the other hand, the main down-regulated metabolites were: cystine, aspartylphenylalanine, leucine, aminobutyric acid, decanoylcarnitine, 3-carboxy-4-methyl-5-pr, carnitine, nicotinamide riboside, pentachlorophenol, coniferyl alcohol, 3-3,4,5-trimethoxyphen and abscisic acid. Compared to healthy individuals, creatine was significantly upregulated in FESCZ patients, while betaine, nonanoic acid, benzoic acid and perillic acid were significantly downregulated. The metabolic profile of pituitary tissue in patients with SCZ was also altered. Compared to healthy individuals, SCZ patients showed significantly upregulated levels of fibrinogen beta chain, proopiomelanocortin, and myosin-9 in their pituitary tissue, while levels of prolactin, secretagogin, catenin delta-2, transglutaminase 2, apolipoprotein A2, tubulin beta chain, and alpha-2-hs-glycoprotein were significantly downregulated. Compared to healthy individuals without toxoplasma infection, SCZ patients with concurrent toxoplasma infection showed a significant increase in α-hydroxyglutaric acid and caprolactam, while inosine, hypoxanthine, and xanthine were significantly downregulated. Effective treatment can reverse metabolic changes. After treatment, patients with SCZ showed a significant increase in palmitic acid, phenylalanine, tyrosine, uric acid, and γ-tocopherol, and a significant decrease in androsterone, aspartate, glucuronic acid, glycine, myo-inositol, and stearic acid. Oleic acid and linoleic acid exhibited upregulation in some studies and downregulation in others. Comprehensive analysis revealed that tyrosine and γ-tocopherol were downregulated in patients with SCZ and significantly upregulated after effective treatment. Therefore, we speculate that tyrosine and γ-tocopherol can not only be used for the accurate identification of SCZ patients, but also for predicting treatment outcomes.

### 3.6 The predictive potential of metabolite combinations related to SCZ in high-quality literature

A total of 30 studies used metabolites or metabolite combinations to identify SCZ or predict treatment prognosis, among which 8 (16, 27, 35, 44, 47–50) used multiple individual metabolites for AUC analysis, and 22 (6, 13, 14, 19–21, 26, 30, 31, 34, 36, 38, 39, 42, 43, 51–57) used metabolite combinations. At the single metabolite level, methionine sulfoxide had the highest accuracy in identifying Miao SCZ patients (test set AUC = 0.98) and c-glucys had the highest accuracy in identifying general SCZ patients (test set AUC = 0.8874). Pyruvate can be used to predict whether SCZ patients will experience auditory hallucinations (AUC=0.8394 in test). At the level of metabolite combination, the metabolite group formed by methylamine, dimethylamine, N-(1-deoxy-1-fructosyl) isoleucine, phenylalanylphenylalanine, LPA (18:1 (9Z)/0:0), and oleamide showed the best accuracy in identifying SCZ patients (training set AUC=1, test set AUC=1), thencholic acid, 4,8 dimethylnonanoyl carnitine, 3-hydroxycapric acid and prostaglandin A2 also showed good performance (training set AUC=0.9917, test set AUC=0.9945). The metabolite panel consisting of PC 32:1, Pe 34:2, PE (O-34:3), and aspartic acid performed well in identifying both untreated SCZ and medically treated SCZ patients (training set AUC = 0.936, test set AUC = 0.963). Myoinositol, uric acid, and tryptophan can serve as a metabolite combination to predict the treatment efficacy in SCZ (test set AUC=0.949). Imidazolepropionic acid, erythronic acid, homoserine, and aspartic acid can be used to predict the salivation induced by chlorpromazine treatment in SCZ (test set AUC=0.791).

### 3.7 Summary of metabolic pathways associated with SCZ

**Figure 5:**
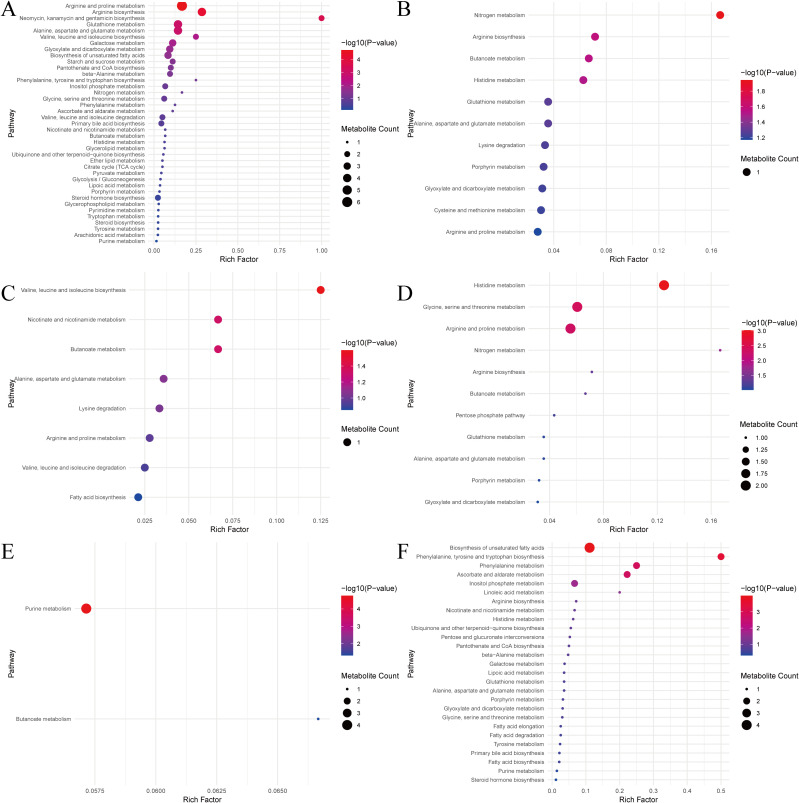
Enrichment results of metabolites pathways in high-quality literature. The size of the circle represents the number of metabolites, and the larger the circle, the more metabolites are enriched. The color of the circle represents the significance of the enrichment analysis. The redder the color of the circle, the more significant the enrichment analysis. **(A)** Schizophrenia versus healthy control. **(B)** Miao ethnic schizophrenia versus Miao ethnic healthy control. **(C)** Miao ethnic schizophrenia versus Han ethnic schizophrenia. **(D)** First-episode schizophrenia versus healthy control. **(E)** Schizophrenia patients co-infected with toxoplasmos versus healthy control not infected with toxoplasmosis. **(F)** Schizophrenia patients at baseline versus schizophrenia patients after intervention.

We conducted metabolic pathway enrichment analysis by matching the metabolites in the high-quality literature with the MetaboAnalyst 6.0 database. This resulted in 6 major categories and 98 metabolic pathways (**Figure 5**). Compared with healthy controls, there were significant enrichment pathways in SCZ such as arginine and proline metabolism, arginine biosynthesis, neomycin, kanamycin and gentamicin biosynthesis, glutathione metabolism, alanine, aspartate and glutamate metabolism, valine, leucine and isoleucine biosynthesis, galactose metabolism, glyoxylate and dicarboxylate metabolism, biosynthesis of unsaturated fatty acids, starch and sucrose metabolism, pantothenate and CoA biosynthesis, beta-alanine metabolism and other pathways. In the comparison between ethnic Miao and Han SCZ patients in China, the metabolite pathway enrichment analysis identified pathways such as nitrogen metabolism, arginine biosynthesis, butanoate metabolism, and histidine metabolism. Valine, leucine and isoleucine biosynthesis, nicotine and nicotinamide metabolism, butanoate metabolism and other pathways were significantly enriched in the comparison between SCZ patients and the healthy control population within the Miao ethnic group in China. Pathways such as histidine metabolism, glycine, serine and threonine metabolism, argenine and proline metabolism, nitrogen metabolism, argenine biosynthesis, and butanolate metabolism were significantly enriched in comparison between FESCZ patients and the healthy controls. Only the purine metabolism and butanoate metabolism pathways were significantly enriched in the comparison between SCZ patients co-infected with toxoplasmos and healthy people not infected with toxoplasmosis. The biosynthesis pathways of unsaturated fatty acids, phenylalanine, tyrosine, and tryptophan, phenylalanine metabolism, ascorbate and aldarate metabolism, inositol phosphate metabolism, and linoleic acid metabolism were significantly enriched in the comparison before and after effective treatment.

## 4 Discussion

### 4.1 Research implications

In this study, different metabolites in serum, plasma, cerebrospinal fluid (CSF), saliva, prefrontal cortex and pituitary tissues were found between SCZ patients and healthy controls, such as arachidonic acid, GABA, tryptophan and proline. These metabolites are involved in the pathogenesis of SCZ through a variety of metabolic pathways, including glutathione metabolism, butanoate metabolism, histidine metabolism, linoleic acid metabolism and kynurenine pathway, involving various biological mechanisms, such as oxidative stress response, energy metabolism, purine metabolism. This study has facilitated the deepening of clinical staff’s understanding of the pathogenesis of SCZ. At the same time, it has provided clinical staff with a potential diagnostic biomarker combination and provided options for subsequent accurate diagnosis.

### 4.2 Comparison to previous studies

A systematic review of body fluid metabolomics for SCZ patients analyzed the differential metabolites and metabolic pathways between SCZ patients and healthy subjects (5), the study found that the content of polyunsaturated fatty acid (linoleic acid) and creatinine decreased in SCZ patients, which is consistent with the results of our study. In our study, in addition to the literature on serum, plasma, CSF, and peripheral blood mononuclear cells, we also selected studies involving the prefrontal cortex and pituitary gland. Thus, the specimens in this study are both more comprehensive and more convincing. Lipidomics, as a branch of metabolomics, can also be used to study the differences in metabolites and metabolic pathways between patients and healthy controls. A recent systematic review, including brain tissue (prefrontal cortex) and peripheral blood, analyzed lipidomic changes in SCZ patients from four perspectives: triglycerides, phospholipids, sphingolipids, and steroids (67). The study found that the levels of sulfates, N-acylphosphotidylserines, ethanolamine, and choline plasminogen were significantly upregulated in brain tissue, while the levels of phosphatidylethanolamine, lysophosphatidylethanolamine, phosphatidylcholine, lysophosphatidylcholine, and ethanolamine plasminogen were significantly downregulated in peripheral tissues. Moreover, the level of ethanolamine plasmalogen was significantly down-regulated in the plasma of patients with both first-episode and recurrent SCZ, which is crucial for clinical diagnosis. In our study, we also revealed that the plasma levels of phosphatidylethanolamine and lysophosphatidylcholine had decreased in women with SCZ. Moreover, we found that isocitrate, succinic acid and itaconic acid were up-regulated, and L-3,4-dihydroxyphenylalanine (L-dopa), dopamine 3-osulfate and norepinephrine sulfate were down-regulated in FESCZ patients. Another study has shown abnormal metabolism of isoform 1 of dimethylarginine dimethylaminohydrolase (DDAH1) and arginase in patients with SCZ, manifested specifically by abnormally elevated levels of asymmetric dimethylarginine (ADMA), dimethylamine, and ornithine (68), However, the study found no significant difference in arginine levels between SCZ patients and controls. Ourstudy not only revealed that ADMA and ornithine increased in SCZ patients, but also that ornithine can be used to predict whether cognitive function of SCZ patients is impaired, and L-ornithine and D-ornithine decreased in SCZ patients. In contrast to previous studies, we also found that arginine levels decreased in patients with SCZ, but the results for L-arginine levels were not consistent across studies. Several systematic reviews have indicated that omega-3 dietary supplementation can mitigate negative symptoms in FESCZ and partially alleviate the symptoms of CSCZ patients (69). Our study also indicated that the content of omega-6 polyunsaturated fatty acids (PUFA) increased in patients with SCZ. Therefore, we speculate that different types of fatty acids may play distinct roles in the pathogenesis of SCZ. More and more studies are exploring the role of the kynurenine pathway in the pathogenesis of SCZ. It is generally believed that the kynurenic acid (KYNA) content increases in the central nervous systems (CNS) of SCZ patients, and that the kynurenine pathway is over-activated in SCZ patients (70). However, in our study, we found that KYNA levels had declined in peripheral serum, which may have been due to the inability of KYNA to penetrate the blood-brain barrier. The above comparison further confirms the role of specific metabolites in the diagnosis and prognosis of SCZ.

### 4.3 Mechanism analysis

A single study may be affected by confounding factors, making it difficult to derive strong persuasiveness. Therefore, in this systematic review, we comprehensively analyzed multiple studies and found that the changes from metabolites to pathways further revealed the pathological mechanism of SCZ at the metabolite level.

Amino acids are known for supporting neural function in two ways, directly acting as neurotransmitters to mediate neural communication and participate in signal transmission in neurons, and indirectly participating in energy supply as metabolic substrate (71). It is generally believed that abnormal arginine metabolism is involved in the pathogenesis of SCZ from multiple perspectives, such as NO regulation, immune inflammatory response, and mitochondrial dysfunction. Previous studies have found that the arginine biosynthetic pathway is a genetic risk factor for SCZ (51), and our study also found significant enrichment in SCZ versus healthy populations. Arginine can be decomposed into many metabolites under different conditions: nitric oxide (NO), citrulline, ornithine, urea and agmatine. Arginine is a precursor substance for the synthesis of NO. The gaseous signaling molecule NO plays an important role in nerve signaling and nerve cell protection, and can also regulate synaptic plasticity, neurodevelopment and cerebral blood flow. Excessive free radical NO can produce neurotoxicity and neurodegeneration (72). NO synthesis is affected in SCZ and may be related to changes in arginine metabolites. For example, the levels of ADMA and ornithine vary significantly in SCZ, and changes in these metabolites may affect the synthesis of NO and thus the functional and behavioral performance of neurons (67). Ornithine can be directed to the production of glutamic acid, glutamine, and GABA (73), and is a major precursor to polyamine putrescine, spermidine, and spermine, which are essential for maintaining normal cellular function. Immune inflammation is considered an important factor in the pathogenesis of SCZ. The levels of inflammatory factors (such as IL-6) are significant in patients with SCZ, and arginine metabolism is closely related to the inflammatory response. Arginine regulates the inflammatory response by affecting the activity of microglial cells, which may alleviate neuronal damage and the exacerbation of psychiatric symptoms (74). Mitochondrial dysfunction is closely related to the pathogenesis of SCZ. Changes in arginine metabolism may affect mitochondrial energy metabolism (75), leading to insufficient energy and oxidative stress in neurons, increasing the risk of SCZ.

Arginine metabolism can regulate tryptophan metabolism. Arginine can activate mTOR signaling pathway, and mTOR activation can regulate the activity of indoleamine-2,3-dioxygenase (IDO). IDO is a key enzyme in tryptophan metabolism, which catalyzes tryptophan metabolism along the kynurenine pathway, thus affecting the metabolism level of tryptophan (76). The tryptophan metabolic pathway has been a focus of SCZ researchers. As an essential amino acid, tryptophan is metabolized in the human body primarily through two pathways: the 5-HT pathway and the kynurenine pathway. Approximately 95% of tryptophan is metabolized via the kynurenine pathway. Specifically, tryptophan is converted into kynurenine through the catalytic action of tryptophan-2,3-dioxygenase (TDO) and IDO in the CNS (microglia and astrocytes) and the peripheral nervous system (liver and kidneys). Kynurenine then undergoes further metabolism through two branches: 1) it is converted into KYNA under the action of kynurenine transaminase; 2) it is first converted into 3-hydroxykynurenine (3-HK) under the action of kynurenine monooxygenase (KMO), and then generates 3-amino-4-hydroxybenzoic acid under the action of kynureninase, ultimately breaking it down into quinolinic acid (70). Quinolinic acid and KYNA are agonists and antagonists of N-methyl-D-aspartic acid receptor (NMDAR), respectively, and KYNA is also an α7 nicotinic acetylcholine receptor (α7nAChR) antagonist. Thus, quinolinic acid and KYNA are considered neuromodulators (77). Nicotinic acetylcholine receptors are mainly found in brain regions such as the hippocampus and the prefrontal cortex which regulate cognitive function, as well as in midbrain dopaminergic nerve cells. Additionally, NMDAR can negatively regulate the uptake and release of glutamate in the presynaptic membrane. Therefore, the abnormally high content of KYNA can dysregulate multiple neurotransmitters, such as glutamate, dopamine and acetylcholine (78–81). Research has indicated that abnormally elevated levels of KYNA are associated with cognitive function impairment, suggesting that the aforementioned neurotransmitter may mediate this pathological process (77).

Other than arginine metabolism, proline metabolism is the most significant pathway for functional enrichment in SCZ (82). Proline is a nonessential amino acid synthesized by pyrroline-5 carboxylate synthesis (P5CS) and pyrroline carboxylate reductases (PyCRS), and decomposed by proline dehydrogenase (PRODH) (83). It is involved in many important metabolic processes, such as glycolysis, the tricarboxylic acid cycle and the pentose phosphate pathway. PRODH is the first proline-degrading enzyme in the metabolic pathway, and it has been identified as a susceptibility gene for SCZ (84). Animal studies confirm that PRODH knockout mice exhibit SCZ-like behavior (85), We speculate that this may be because reduced proline degradation causes accumulation, and proline accumulation can independently lead to the onset of SCZ. This may be because proline has structural similarities with GABA and glutamate, which may affect the binding site (86).

Modern neuroimaging research has shown that the first brain area affected in patients with SCZ is the hippocampus, and specifically its CA1 area (87). The main pathophysiological mechanisms of hippocampal dysfunction are hyperactivity, atrophy, and abnormal increase in glutamate content (88). However, the level of glutamate in SCZ-related studies has historically been controversial (89). Recent research suggests that primary pyramidal dysfunction leading to decreased glutamate levels is at the forefront of SCZ studies (90). Our research reveals new insight into the changes in glutamate associated with SCZ. It suggests that the manifestation of positive symptoms in SCZ also depends on secondary disinhibition effects, which arise from downstream adaptive changes in inhibitory feedback, resulting in an increase in glutamate levels compared to previous states. Specifically, the full process of SCZ is divided into three stages: the genetic susceptibility state, the clinical high-risk state, and the diagnosed state with symptomatic manifestations. People who are genetically susceptible have lower levels of glutamate, especially in the anterior cingulate cortex. The low levels of glutamate in the population are associated with the core symptoms of SCZ, reflecting reduced excitability; people at high clinical risk will have a secondary disinhibition effect, and their levels of glutamic acid will be relatively excessive, and positive symptoms will also appear. When the deinhibition state is fully present, the patient will exhibit significant core symptoms, and glutamate levels will normalize (90). A recent clinical trial has revealed similar results for glutamate content in the anterior cingulate cortex by comparing glutamate patients with genetically high risk, clinically high risk, FESCZ (91). This theoretical interpretation profoundly explains the broad variation inglutamate content in patients, and why the linear relationship between glutamate content and core symptoms of SCZ is elusive.

The pathogenesis of SCZ is related to multiple metabolites, and some patients may also have sleep disorders. Research has shown that butanoate metabolism plays an important role in the pathogenic mechanism of sleep disorders in patients with SCZ (92). Another study revealed that butanoate metabolism was significantly downregulated in the gut microbiomes of patients after amisulpride treatment (93). Our study found that butanoate metabolism was significantly enriched in Chinese ethnic Miao patients, FESCZ, patients with SCZ and toxoplasma infection. This shows that butanoate metabolism is widely involved in the pathogenesis of SCZ, yet the current understanding of butanoate metabolism in the field of neuropsychiatry is not comprehensive. Thus, its role remains to be explored. Butanoate metabolism is significantly downregulated in patients with Parkinson’s disease (94), and is also significantly associated with prenatal depression (95). In metabolomics studies, butyric acid metabolism has been considered a form of carbohydrate metabolism. We speculate that the correlation between SCZ and butyric acid metabolism may be mainly due to the downregulation of butyric acid metabolism leading to energy supply disorders in patients with SCZ.

Our study is generally consistent with the key pathological mechanisms (oxidative stress, neuroinflammation, energy metabolism) discovered in previous studies (5). In fact, there are many metabolites closely related to the pathological mechanisms of SCZ, such as lipid metabolites: LPC (18:0), LPC (20:0), PC (18:2/18:2), PC (O-16:0/18:2), LPE (20:4), and PE (P-18:0/18:2), which collectively improve the diagnostic accuracy of SCZ (39). As a long-chain organic acid, fatty acid metabolism plays an important role in the pathogenesis of SCZ. PUFA are important fatty acids which can be classified as either ω-3 fatty acids or ω-6 fatty acids. Linoleic acid and linoleic acid are ω-3 fatty acids and ω-6 fatty acids, respectively, which affect the energy supply of SCZ (13, 14, 46, 50). Arachidonic acid is another important ω-6 fatty acid, and it has the most consistent findings in SCZ considering that the content of arachidonic acid is decreased in patients with SCZ (5, 46, 50).

In summary, the multiple metabolites we found in this study not only summarize the metabolic disorders related to SCZ, but also indicate the direction for effective treatment. Metabolites are widely involved in the pathogenesis of SCZ and are also effective indicators for judging and predicting treatment effectiveness.

### 4.4 Challenges in current research

There has been considerable progress in metabolomics research in both experimental design and metabolite detection techniques. However, the current research results cannot fully explain the pathological mechanism of the disease, and many challenges remain in this field. Firstly, the current trial designs primarily focus on case-control studies, exploring the metabolic changes in patient populations by comparing them with healthy individuals. This comparative approach is beneficial for uncovering the pathological mechanisms of diseases at the metabolic level. At present, the case-control design of SCZ has been well studied, and we suggest that more metabolomics studies be placed on clinical treatment to explore the mechanism of effect from the metabolomics level. Secondly, considering the convenience of sampling, most of the current research analyzes the changes of metabolites in the serum and plasma, and the changes of metabolites in the peripheral circulation system cannot completely reflect the changes of metabolites in CNS. Thus, it has been suggested that the changes of metabolites in CSF and brain tissue deserve more attention, and that the changes of metabolites in both central and peripheral tissues should be considered jointly in subsequent research. Finally, due to its genetic specificity, SCZ is the result of the interaction between genetic and environmental factors, and current research has under-emphasized impoverished regions, such as Africa. In order to fully understand the pathogenesis of SCZ in different populations, patients from impoverished regions such as Africa should also be studied.

## 5 Conclusions

In this study, we have summarized the current clinical metabolomics research progress related to SCZ, and thoroughly explored the metabolite changes related to onset and prognosis among different populations. This systematic review summarizes dozens of studies, deepening our understanding of the pathogenesis of SCZ and laying the foundation for future research on metabolites related to SCZ.

## Data Availability

The original contributions presented in the study are included in the article/**Supplementary Material**. Further inquiries can be directed to the corresponding authors.
