# Supplementary material for: Discovery of biological markers for schizophrenia based on metabolomics: a systematic review
Source: Front Psychiatry. 2025 Mar 28;16:1540260. doi: 10.3389/fpsyt.2025.1540260 (PMC11985778; doi:10.3389/fpsyt.2025.1540260)
Supplement: Supplementary file 1 [file DataSheet1.zip › Supplementary materials/Search strategy.docx]

**Search strategy:**

**PubMed:**

#1 ((((((Schizophrenia[MeSH Terms]) OR (Schizophrenias[Title/Abstract])) OR (Dementia Praecox[Title/Abstract])) OR (Schizophrenic Disorders[Title/Abstract])) OR (Disorder, Schizophrenic[Title/Abstract])) OR (Disorders, Schizophrenic[Title/Abstract])) OR (Schizophrenic Disorder[Title/Abstract])

#2 ((metabolomics[MeSH Terms]) OR (metabolome[MeSH Terms])) OR (metabolic flux analysis[MeSH Terms])

#3 ((((((((((((Metabolomic[Title/Abstract]) OR (Metabonomics[Title/Abstract])) OR (Metabonomic[Title/Abstract])) OR (Metabolomes[Title/Abstract])) OR (Metabolic Profile[Title/Abstract])) OR (Metabolic Profiles[Title/Abstract])) OR (Profile, Metabolic[Title/Abstract])) OR (Profiles, Metabolic[Title/Abstract])) OR (Analyses, Metabolic Flux[Title/Abstract])) OR (Analysis, Metabolic Flux[Title/Abstract])) OR (Flux Analyses, Metabolic[Title/Abstract])) OR (Flux Analysis, Metabolic[Title/Abstract])) OR (Metabolic Flux Analyses[Title/Abstract])

#4 #2 OR #3

#5 #1 AND #4

#6 ((review[Title]) OR (systematicreview[Title])) OR (meta analysis[Title])

#7 #5 NOT #6

**Embase:**

#1 'schizophrenia'/exp

#2 'schizophrenia':ab,ti OR 'childhood schizophrenia':ab,ti OR 'chronic schizophrenia':ab,ti OR 'dementia praecox':ab,ti OR 'dementia precox':ab,ti OR 'schizophrenia, childhood':ab,ti OR schizophrenic:ab,ti OR 'schizophrenic language':ab,ti OR 'schizophrenic syndrome':ab,ti

#3 #1 OR #2

#4 'metabolomics'/exp

#5 'metabolomics':ab,ti OR 'metabolome':ab,ti OR 'metabolic flux analysis':ab,ti OR 'metabonomics':ab,ti OR 'metabonomic':ab,ti OR 'metabolic fingerprinting':ab,ti

#6 #4 OR #5

#7 #3 AND #6

#8 'review':ab,ti OR 'systematic review':ab,ti OR 'meta analysis':ab,ti

#9 #7 NOT #8

**WOS:**

#1 TS=(Schizophrenia) OR TS=(Schizophrenias) OR TS=(Dementia Praecox) OR TS=(Schizophrenic Disorders) OR TS=(Disorder, Schizophrenic) OR TS=(Disorders, Schizophrenic) OR TS=(Schizophrenic Disorder)

#2 TS=(metabolomics) OR TS=(metabolome) OR TS=(metabolic flux analysis) OR TS=(metabolic profiling) OR TS=(metabolic signature) OR TS=(metabolic biomarker) OR TS=(meta-bolic profile) OR TS=(Metabolomic) OR TS=(Metabonomics) OR TS=(Metabonomic) OR TS=(Metabolomes) OR TS=(Metabolic Profile) OR TS=(Metabolic Profiles) OR TS=(Profile, Metabolic) OR TS=(Profiles, Metabolic) OR TS=(Analyses, Metabolic Flux) OR TS=(Analysis, Metabolic Flux) OR TS=(Flux Analyses, Metabolic) OR TS=(Flux Analysis, Metabolic) OR TS=(Metabolic Flux Analyses)

#3 #1 AND #2

#4 ((TS=(review)) OR TS=(meta analysis)) OR TS=(systematic review)

#5 #3 NOT #4

#6 #5 AND (Review Article and Editorial Material or Meeting or Letter or Correction or Book or Meeting Abstract or Retracted Publication) (Exclude –Document Types)
