# Supplementary material for: Discovery of biological markers for schizophrenia based on metabolomics: a systematic review
Source: Front Psychiatry. 2025 Mar 28;16:1540260. doi: 10.3389/fpsyt.2025.1540260 (PMC11985778; doi:10.3389/fpsyt.2025.1540260)
Supplement: Supplementary file 1 [file DataSheet1.zip › Supplementary materials/Supplementary Table 1 Table of basic characteristics of included documents.docx]

Supplementary Table 1 Table of basic characteristics of included documents

| Article sources | Country | research subjects | gender | group settings | research type | sample size | analysis platform | research purpose | sample type | Targeted/ non-targeted | Upregulation of metabolites | Downregulation of metabolites | Metabolic pathways |
| --- | --- | --- | --- | --- | --- | --- | --- | --- | --- | --- | --- | --- | --- |
| Okamoto,2021 | Japan | CSCZ | ALL | CSCZ(30)  HC(10) | Case control | 40 | CE-MS | clinical diagnosis | serum | non-targeted | NM | NM | Glutamate metabolism and the urea cycle were decreased in the schizophrenia group. |
| Ye,2024 | China | Han and Miao SCZ | ALL | Han SCZ(30)  Han HC(30)  Miao SCZ(30)  Miao HC(30) | Case control | 120 | LC-MS | clinical diagnosis | serum | non-targeted | glutamate levels in both Miao and Han SCZ, Methionine sulfoxide in Miao SCZ. | Carnitine and Decanoylcarnitine in Miao SCZ. | fatty acid metabolism pathways. |
| Koike,2014 | Japan | FESZ | ALL | FESZ(30)  HC(38) | Case control | 68 | CE-MS | clinical diagnosis | plasma | non-targeted | creatine | betaine,nonanoic acid, benzoic acid and perillic acid | NM |
| Liu,2021 | China | SCZ | ALL | Drug Naïve SCZ(38)  HC(25)  Drug-treated SCZ(38)  HC(25) | Case control | 126 | LC/MS/MS | clinical diagnosis | plasma | targeted | LPC 16:0 sn2, LPE 16:1 sn1, LPE 16:1 sn2, LPE 22:5 sn2, PC 32:1, PE 34:2 | Aspartic acid, Carnitine C10:1, Carnitine C10:2, Carnitine C8:1, FFA 16:2, FFA 18:2, FFA 18:3, FFA 20:4, FFA 22:6, Lithocholic acid, PC (O-34:2), PE (O-34:3), PE (O-36:6) | tRNA Charging, Superpathway of Citrulline Metabolism, Citrulline Biosynthesis, Proline Biosynthesis II (from Arginine), Asparagine Biosynthesis I, Arginine Biosynthesis IV, Urea Cycle |
| Wang,2018 | China | SCZ | ALL | SCZ(115)  HC(108) | Case control | 223 | LC-MS | clinical diagnosis | serum | targeted | 11,12-DHET, 14,15-DHET,20-carboxy-AA,15-HETE,8-HETE,12-HEPE,AEA,OEA,15-KEDE | AA,PGE2, PGA2,PGF2α,PGJ2,TXB2, 11-dehydro-TXB2, LTB4,5-KETE,12-HpETE,12-HETE, 11-HETE,DHA,13-HdoHE, EPA | Cyclooxygenase, cytochrome P450, lipoxygenase, non-enzymatic, |
| Schwarz,2011 | UK | SCZ | ALL | Drug-treated SCZ(70)  HC(59) | Case control | 129 | LC-MS | clinical diagnosis | serum | targeted | Oleamide, Heptadecenoic amide, Palmitic amide, Palmitoleic amide, Myristic amide | NM | NM |
| Liu,2016 | New Zealand | SCZ | ALL | SCZ(20)  HC(20) | Case control | 40 | HPLC | clinical diagnosis | Prefrontal cortex | targeted | glutamate/GABA ratio | GABA, agmatine | NM |
| Yao,2010 | USA | SCZ | ALL | SCZ(25)  HC(30) | Case control | 55 | LC | clinical diagnosis | plasma | targeted | Nacetylserotonin , melatonin (Mel)/serotonin ratios ,NA5HT and the ratio of NA5HT to its precursor (Trp) | ratio of Mel (metabolite) to NA5HT | serotonin, kynurenine and tryptamine pathways |
| Mednova,2021 | Russia | SCZ | ALL | SCZ(37)  HC(36) | Case control | 73 | MS | clinical diagnosis | plasma | NM | C4-DC | Valine,aspartate,citrulline,glycine,arginine,ornithine. longchain species: C14,C14-OH,C16-OH,C16:1,C16:1-OH,C18,C18-OH,C18:1,C18:1-OH,C18:2-OH,and short-chain acylcarnitine C5:1, | NM |
| Wang,2022 | China | SCZ | ALL | SCZ(64)  HC(40) | Case control | 104 | LC-MS and 1H NMR | clinical diagnosis | plasma | non-targeted | NM | NM | disorder of lipid metabolism, amino acid metabolism, and energy metabolism might be the major pathogenic factors of schizophrenia |
| Costa,2023 | Brazil | SCZ | ALL | SCZ(30)  HC(30) | Case control | 60 | LC-MS | clinical diagnosis | plasma | non-targeted | glycerophospholipids (GP), particularly LPC 16:0, sphingolipids (SP), such as glycosphingolipids and ceramides, several types of sterol lipids (ST), and fatty acids (FA) | SP, predominantly sphingomyelins; glycerolipids (GL), such as triacylglycerols, as well as some species of GP and ST | androgen and estrogen biosynthesis and metabolism, bile acid biosynthesis, GP metabolism,glycosphingolipid (GSP) biosynthesis—globo series, GSP metabolism, linoleate metabolism,phosphatidylinositol phosphate metabolism, GSP biosynthesis—ganglio series |
| Liu,2022 | China | cognitively impaired SCZ, cognitively normal SCZ | ALL | cognitively impaired SCZ(15), cognitively normal SCZ(15) | cross-sectional study | 30 | LC | Complications identification | serum | non-targeted | NM | sphinganine and 3-dehydrosphinganine, D-glutamine,pyrrolidonecarboxylic acid, choline and creatine | sphingolipid metabolism ,D-glutamine and D-glutamate metabolism,glycine, serine and threonine metabolism |
| Krzyściak,2024 | Poland | SCZ | ALL | SCZ(51)  HC(45) | Case control | 96 | LC- MS | clinical diagnosis | serum | non-targeted | NM | cortisol, glutamic acid, and lactates | NM |
| Ward,2018 | USA | SCZ | ALL | SCZ(94)  HC(20) | Case control | 114 | GC | clinical diagnosis | serum | non-targeted | NM | NM | one-carbon metabolism pathway |
| Wang,2021 | China | SCZ | ALL | SCZ(119)  HC(109) | Case control | 228 | LC-MS | clinical diagnosis | serum | non-targeted | saturated fatty acids (SFAs), monounsaturated fatty acids (MUFA), and polyunsaturated fatty acids (PUFAs), LPCs with PUFA residues C20:4 and C22:6, LPEs with PUFA | LPCs with SFA or MUFA side chain, | NM |
| Cao,2019 | China | SCZ | ALL | SCZ(225)  HC(175) | Case control | 400 | LC-MS | clinical diagnosis | plasma | targeted | C4-OH (C3-DC) and C16:1 | C3, C8, C10, C10:1, C10:2, C12,C14:1-OH, C14:2, and C14:2-OH | NM |
| Cao,2018 | China | SCZ | ALL | SCZ(208)  HC(175) | Case control | 383 | LC-MS | clinical diagnosis | plasma | non-targeted | cysteine, GABA, glutamine, and  sarcosine | arginine, l-ornithine, threonine, taurine, tryptophan, methylcysteine, and  kynurenine | NM |
| Ji,2021 | Australia | SCZ | ALL | SCZ(94)  HC(81) | Case control | 175 | LC-MS | clinical diagnosis | serum | targeted | DHEA | Cortisol/DHEA ratios | NM |
| Bicikova,2013 | Czech | SCZ | ALL | SCZ(22)  HC(22) | Case control | 44 | GC-MS | clinical diagnosis | serum | non-targeted | pregnenolone sulfate and sulfated 5α- as well as 5β-saturated metabolites of C21-steroids | 5α- metabolites of C21 steroids, 5β-reduced metabolites | progesterone metabolic pathway |
| Chen,2020 | China | V.SC | ALL | V.SC(53)  NV.SC(24) | cross-sectional study | 77 | GC-MS | Complications identification | plasma | non-targeted | Vanillylmandelic acid, Glycerol, Glyceraldehyde | Malic acid,4-Hydroxyphenylpyruvic acid,4-Hydroxy-L-proline,L-Methionine,Ratio of L-asparagine/  L-aspartic acid, Malonic acid, Glycerol 3-phosphate, D-Ribose,Uric acid,3-Aminoisobutanoic acid,L-Sorbose,3-Aminosalicylic acid, Glutaric acid,4-Hydroxyproline,Ribitol,Ribonolactone | Amino acid metabolism, Lipid metabolism,Pentose phosphate pathway,Purine metabolism,Pyrimidine metabolism |
| Liu,2014 | China | SCZ | ALL | SCZ(45)  HC(50) | Case control | 95 | GC-MS | clinical diagnosis | Peripheral blood monocytes | targeted | hydroxylamine,octanoic acid, glycerol, aspartic acid, 2-hydroxyethyl almitate, benzoic acid and homoserine | methyl phosphate, valine, fumaric acid, pyroglutamic acid, creatinine, sorbitol, inositol, dopamine, maltose, tocopherol-γ and tocopherol-α | energy metabolism, oxidative stress and neurotransmitter metabolism |
| Zhang,2023 | China | CIS | ALL | CIS (32)  NOT CIS (19) | Case control | 51 | LC-MS | Complications identification | saliva | non-targeted | guanosine, adrenaline, pyroglutamate, histidine, ribitol, deoxycytidine, histamine, dihydrouracil, cytosine, dopamine, 5-hydroxytryptophan, thymine, 5-hydroxylysine, 1-methylhistamine,3-methylhistamine, pyrimidine, and glycyl-tyrosine | alliin, D-ornithine, isoleucyl-glutamate, hypoxanthine, kynurenine, herniarin, uridine, N-methyl-proline, tyramine,indolepyruvate, flavin mononucleotide, kinetin, phloretin, γ-glutamylleucine, N-acetyl-L-methionine, lysine,D-Proline, 5,7-dihydroxyisoflavone, aspartic acid, and guanine | Histidine metabolism, pyrimidine metabolism, and β-alanine metabolism |
| Su,2023 | China | SCZ | ALL | SCZ(60)  HC(36) | Case control | 96 | LC-MS | clinical diagnosis | plasma | targeted | CA,CE(16:1),ProBetaine,Cortisol,CE(15:0),Sarco sine | GABA,C2,PCaa C36:0,Cer(d18:1/24:0),PC ae C34:0, Cer(d18:1/22:0),PCaaC36:2,PCae C38:2, PCae C34:3,PC ae C40:1,PCae C36:2, PC aa C40:2, PCaeC36:3,Choline,PCae C36:4, SM(OH) C22:1, Spermidine,PCaa C42:2,PCaeC36:5,lysoPCa C18:0 | NM |
| Yin,2024 | China | SCZ | ALL | SCZ(37)  HC(50) | Case control | 87 | LC-MS | clinical diagnosis | plasma | non-targeted | Pyroglutamic acid | 3-methylcytidine, Adenosine 3',5'-cyclic phosphate (cAMP),  Car(11:1)_RT383,  Car(16:2-O)_RT395,  Car(9:0)_RT346, Xanthurenic acid | amino acid metabolism, lipid metabolism, nucleotide metabolism, carbohydrate metabolism, metabolism of cofactors and vitamins |
| Fujii,2017 | Japan | SCZ | ALL | SCZ(29)  HC(30) | Case control | 59 | CE-MS | clinical diagnosis | frontal cortex and Hippocampus | non-targeted | frontal cortex: Glucose 6-phosphate, Gly-Gly (glycylglycine), Diphenylcarbazide, Gluconic acid; Hippocampus: Gly-Gly (glycylglycine), Lactic acid, Pyridoxamine | frontal cortex: Mevalolactone, 3-Amino-2-piperidone, Adenylosuccinic acid; | Central carbon metabolism in cancer, Protein digestion and absorption,Aminoacyl-tRNA biosynthesis, ABC transporters, Mineral absorption, Cyanoamino acid metabolism,Alanine, aspartate and glutamate metabolism,Glycolysis/Gluconeogenesis |
| Cao,2020 | China | SCZ | ALL | SCZ(113)  HC(111) | Case control | 224 | LC-MS | clinical diagnosis | serum | non-targeted | Oleoylcarnitine,L-Palmitoylcarnitine,  Tauri | 9-Decenoylcarnitine,  2-trans,4-cis-Decadienoylcarnitine,LPC(P-16:0), LPC(16:0), LPC(15:0), LPC(14:0), 2,5-Dichloro-4-oxohex-2-enedioate, L-Arginine | fatty acid metabolism, amino acid metabolism |
| Wang,2019 | China | SCZ | ALL | SCZ(119)  HC(109) | Case control | 228 | LC-MS | clinical diagnosis | serum | non-targeted | LysoPC(22:6), LysoPE(16:0), LysoPE(20:4),  PC(15:0/18:1),  PC(16:0/16:0),  PC(16:0/18:1),  PC(16:0/22:5),  PC(16:1/16:0), PC(20:4/18:0), SM(d18:0/18:1),  SM(d18:0/18:2),  SM(d18:1/16:0) | LysoPC(14:0),  LysoPC(15:0),  LysoPC(17:0),  LysoPC(17:1),  LysoPC(18:0),  LysoPC(0:0/18:0),  LysoPC(19:0),  LysoPC(20:0),  LysoPC(20:1), LysoPE(20:0), PC(18:2/17:0),  PC(18:2/17:1),  PC(18:2/18:2),  PC(20:3/16:0),  PC(20:4/14:0), PC(20:4/18:2),  PC(22:6/14:0),  PC(O-16:0/18:2),  PC(O-16:0/20:3),  PC(O-16:0/20:4),  PC(O-18:0/20:4),  PC(O-18:0/22:6),  PC(P-16:0/18:2) | NM |
| Holmes,2006 | Germany | SCZ | ALL | SCZ(37)  HC(70) | Case control | 107 | NMR | clinical diagnosis | cerebrospinal fluid | non-targeted | glucose | Acetate, lactate | NM |
| Krishnamurthy,2013 | Germany | SCZ | ALL | post-mortem SCZ(14)  CON(15) | Case control | 29 | LC-MS | clinical diagnosis | pituitary | non-targeted | Signal-induced proliferation-associated protein 1,  Protein  Fibrinogen beta chain, WD repeat-containing protein C10orf79,  Myosin-9 | Prolactin, Vasopressin-neurophysin 2-copeptin (AVP-NP2 precursor), Secretagogin | NM |
| Orešič,2011 | Finland | SCZ | ALL | SCZ(45)  HC(45) | Case control | 90 | LC-MS | clinical diagnosis | serum | non-targeted | branched chain amino acids, phenylalanine, tyrosine, proline, glutamic,lactic pyruvic acids | Acetoacetic acid, beta-hydroxybutyric acid, stearic acid, oleic acid | NM |
| Du,2021 | China | SCZ | ALL | SCZ(78)  HC(66) | Case control | 144 | LC-MS | clinical diagnosis | serum | targeted | amino acid l-arginine | glycerophospholipid | glycerophospholipid metabolism and the biosynthesis of phenylalanine, tyrosine, and tryptophan |
| Osman,2022 | Malaysia | SCZ | ALL | TP+Sz(15) TN+Sz(15)  TP+NSz(15) TN+NSz(15) | Case control | 60 | LC-MS | clinical diagnosis | plasma | non-targeted | α-hydroxyglutaric acid, Caprolactam | 3,30-Thiopropionic acid, adenosine monophosphate, inosine, hypoxanthine and xanthine | NM |
| Xuan,2011a | China | SCZ | ALL | SCZ(18)  HC(18) | Case control | 36 | GC-MS | clinical diagnosis | serum | non-targeted | Glucose, Lactate, Allantoin, Myo-inositol,  Glucuronic acid, Glycerol,  Cholesterol,  Lactobionic acid,  Erythrose | 1,3-Bisphosphoglycerate, Citrate,  α-Ketoglutarate, Uric acid,  γ-Tocopherol,  N-Acetylaspartate,  Aspartate,  Glycine,  Tryptophan, Linoleic acid,  Oleic acid,  Stearic acid,  Palmitic acid, | Glycolysis,TCA Cycle, Uric acid metabolism,  Purine metabolism,  Vitamin E metabolism,  Alanine, aspartate and glutamate metabolism, Glycine, serine and threonine metabolism,  Tryptophan Metabolism,  Inositol phosphate metabolism, Fatty acid metabolism,  Glycerolipid metabolism,  Steroid biosynthesis |
| Xuan,2011b | China | SCZ | ALL | Pre-SCZ(18)  Pos-SCZ(18) | 前后自身对照 | 36 | GC-MS | prognosis | serum | non-targeted | Phenylalanine,  Tyrosine, Linoleic acid, Palmitic acid, Oleic acid, Tryptophan, Uric acid, Lactate | Aspartate,  Glycine, Myo-inositol,  Glucuronic acid, Stearic acid, Glycerol, Lactobionic acid,  Erythrose | neurotransmitter metabolism, antioxidant defense systems |
| Li,2022 | China | SCZ | ALL | pAHs(18) nAHs(28) Con(43) | Case control | 89 | LC-MS | Complications identification | serum | targeted | Con and nAHs: valine and cystine,  Con and pAHs: cystine; nAHS vs.pAHs: Phenylalanine, | Con vs. nAHs: phenylalanine, glycerate, hippurate, serine,glutamate and dihydrosphingosine; Con and pAHs: phenylalanine,glycerate, serine, glutamate and dihydrosphingosine; nAHS vs.pAHs: Pyrroline hydroxycarboxylic acid, Pyruvate | D-Glutamine and D-glutamate metabolism, phenylalanine, tyrosine and tryptophan biosynthesis and phenylalanine metabolism |
| Tasic,2017 | Brazil | SCZ | ALL | SCZ(27)  HC(26) | Case control | 53 | ^1^HNMR | clinical diagnosis | serum | non-targeted | pantothenate,mannitol,p-aminobenzoic acid (PABA), glycine, guanine, GABA | L-glutamine and L-threonine, the glycolysis metabolite adenosine, the nucleobase adenine and the isomeric carboxylic acid mesaconic acid | glucose and amino acid metabolism |
| Liu,2020 | China | SCZ | ALL | SCZ(55)  HC(57) | Case control | 112 | ^1^HNMR | clinical diagnosis | serum | non-targeted | Leucine, Proline, Myo-inositol, Pyruvate, Lactate, Creatine, Proline | Lipid (triglycerides and fatty acids), Glutamine,  3-hydroxybutyrate, Dimethylamine,  Citrate, Asparagine, Tyrosine, Glycine, Threonine | alanine, aspartate and glutamate metabolism, TCA cycle, glucose metabolism, and catecholamine metabolism |
| Wang,2022 | China | SCZ | ALL | SCZ(35)  HC(35) | Case control | 70 | LC-MS | clinical diagnosis | serum | non-targeted | N,N-dimethylglycine, S-adenosyl-L-methionine, DL-homocysteine | spermidine | betaine metabolism and methionine metabolism |
| Wang,2024 | China | SCZ | ALL | SCZ(127)  HC(92) | Case control | 219 | LC–MS | clinical diagnosis | serum | non-targeted | neurotoxin Quinolinic acid, Spermidine, | L-2,4-diaminobutyric acid, N(g)-acetyldiaminobutyrate, N-acetylserotonin, Kynurenic acid, Isobutyric acid, Stearicacid, Stearidonicacid | amino acid metabolism, fatty acid metabolism |
| Fan,2022 | China | SCZ | ALL | SCZ(63)  HC(57) | Case control | 120 | LC–MS | clinical diagnosis | serum | non-targeted | asymmetric dimethylarginine,hydroxyisocaproic acid, and 1-methylguanosine | oleic acid, arachidonic acid, alpha-linolenic acid, resolvin D2, and1-methylnicotinamide (MNA) | Oxidative stress metabolites, anti-inflammatory and neuroprotective metabolites |
| Al,2015 | Germany | SCZ | ALL | SCZ(26)  HC(26) | Case control | 52 | GC-MS | clinical diagnosis | serum | non-targeted | 2-Piperidinic carboxylic acid, | 1-Oxo-proline, 6-Deoxy-mannofuranose, Galactose oxime, Oleic Acid, Pentadecanoic acid, Heptadecanoic acid, Eicosanoic acid, Cholesterol | NM |
| Tasic,2019 | Brazil | SCZ | ALL | SCZ(54)  HC(60) | Case control | 114 | NMR | clinical diagnosis | serum | non-targeted | 6-hydroxydopamine (6-OHDA), isovaleryl carnitine, pantothenate, mannitol, glycine, gamma aminobutyric acid(GABA) | N-acetyl-D-mannosamine, 2,3-diphospho-D-glyceric acid, N-acetyl aspartyl-glutamic acid (NAAG), monoethyl malonate | NM |
| Jiang,2022 | China | SCZ | ALL | CI(17) CN(17)  HC(20) | Case control | 54 | LC–MS | Complications identification | plasma | targeted | Lactic acid, 3,4-Dihydroxyhydrocinnamic acid, Aspartic acid, Ornithine, Glutamic acid, Glucose 6-phosphate, N-Methylnicotinamide, Homoserine, Glutamine | Erythronic acid, 2-Furoic acid | Alanine, aspartate and glutamate metabolism, D-glutamine and D-glutamate metabolism, and Citrate cycle |
| Qiao,2016 | China | SCZ | Female | SCZ(15)  HC(15) | Case control | 30 | LC–MS | clinical diagnosis | plasma | non-targeted | l-leucine, phytosphingosine, l-methionine, 2-oxovaleric acid, O-acetylserine, and 2-hydroxyvaleric acid | 3,4,5-trimethoxycinnamicacid, lysophosphatidylcholine (LPC) (20:3), LPC (14:0), phosphatidylethanolamine, citramalic acid, N-acetylglutamic acid, and phosphatidylserine | Lipid metabolism and amino acid metabolism |
| Song,2023a | China | SCZ | ALL | SCZ-BL(43) HC(29) | Case control | 72 | LC-MS | clinical diagnosis | plasma | non-targeted | NM | SCZ-BL: LysoPC, PC, PE, C16 sphinganine, sulfate, dehydroepiandrosterone sulfate (DHEA sulfate), α-tocopherol, vitamin K1, glycocholic acid, taurocholic acid, 1-methylnicotinamide, stearic acid,  behenic acid, α-dimorphecolic, calcidiol, L-tryptophan, hydroxyproline,  and coenzyme Q9. | lipid metabolism, vitamin metabolism, cholesterol metabolism, bile secretion, the biosynthesis of unsaturated fatty acids, and fatty acid metabolism |
| Song,2023b | China | SCZ | ALL | SCZ-BL(43) SCZ-PT(37) | Case control | 80 | LC-MS | prognosis | plasma | non-targeted | SCZ-PT:LysoPC, PC, PE, C16 sphinganine, sulfate,and adrenic acid | SCZ-PT:linoleic acid, oleic acid, palmitoleic acid, γ-linolenic acid, oxoglutaric acid, and androsterone | lipid metabolism, vitamin metabolism, cholesterol metabolism, bile secretion, the biosynthesis of unsaturated fatty acids, and fatty acid metabolism |
| Fukushima,2014 | Japan | SCZ | ALL | SCZ(25)  HC(27) | Case control | 52 | LC | clinical diagnosis | serum | targeted | D-lactate, tryptophan, kynurenine, and glutamate | cglutamylcysteine (c-GluCys), linoleic acid, arachidonic acid, D-serine, 3-hydroxybutyrate, glutathione (GSH), 5-hydroxytryptamine, threonine, and tyrosine | NM |
| Cui,2021 | China | SCZ | ALL | FES(85)  CHR(43)  HC(80) | Case control | 208 | LC- MS | clinical diagnosis | saliva | non-targeted | Isocitrate, succinic acid, itaconic acid, L-2-hydroxyglutarate | L-3,4-Dihydroxyphenylalanine (L-dopa), Dopamine 3-Osulfate and norepinephrine sulfate, normetanephrine | aromatic amino acid metabolism, glutamate metabolism,nucleotide metabolism, tricarboxylic acid cycle |
| He,2012 | Germany | SCZ | ALL | SCZ(265)  HC(216) | Case control | 481 | MS | clinical diagnosis | plasma | targeted | Ornithine | Arginine, Glutamine, Histidine, PC ae C38:6 | Glutamine and arginine metabolism, nitrogen compound biosynthetic process, learning memory behavior |
| Kageyama,2017 | Japan | SCZ BD  MDD | ALL | SCZ(17)  HC(19) | Case control | 36 | CE-MS | clinical diagnosis | plasma | NM | 2-hydroxybutyric acid | Creatine, 2-oxoisovaleric acid | NM |
| Yang,2017 | China | SCZ | ALL | SCZ(110)  HC(109) | Case control | 219 | LC- MS | clinical diagnosis | serum | targeted | Monounsaturated fatty acids (MUFAs) and ω-6 polyunsaturated fatty acids (ω-6 PUFAs) | Very long-chain fatty acid C24:0 | NM |
| Cui,2020 | China | SCZ | ALL | SCZ(54)  HC(54) | Case control | 108 | MS | clinical diagnosis | serum | non-targeted | Phytal  Acetylvalerenolic acid,  Thapsic acid; hexadecanedioic acid,  Ethanolamine oleate,  Pipericine,  Arachidonoyl serinol, Cholesterol, 19-Oxotestosterone | Phenylalanine  Tyrosine,  Tryptophan,  Kynurenine,  Glutamine,  Leucine,  Phenylpyruvate,  o-Tyrosine,  Creatinine, Carnitine,  Acetylcarnitine,  Propionylcarnitine, Glucose, Lactic acid, Triacetin | Phenylalanine, tyrosine, and tryptophan  Biosynthesis,  Steroid hormone biosynthesis,  Nitrogen metabolism,  Glycerophospholipid metabolism,  Arachidonic acid metabolism,  Aminoacyl-tRNA biosynthesis |
| Liu,2015 | China | SCZ  MD | ALL | SCZ(55)  HC(55) | Case control | 110 | GC–MS | clinical diagnosis | Peripheral blood monocytes | targeted | glucose, glucose 6-phosphate, fructose, fructose 6-phosphate, glycerate 3-phosphate, succinic acid, ribose 5-phosphate | glyceraldehyde-3-phosphate, dihydroxyacetone phosphate, glycerol 3-phosphate, citric acid | glucose metabolism |
| Qing,2022 | China | SCZ | ALL | SCZ(108)  HC(108) | Case control | 216 | LC-MS | clinical diagnosis | serum | targeted | apocholic acid (apoCA) and norcholic acid (NorCA), ratio of CA to CDCA | CA,CDCA,DCA, 3β-deoxycholic acid, ursodeoxycholic acid (UDCA) and 3β-chenodeoxycholic acid(βCDCA), 7-ketolithocholic acid (7-ketoLCA) and 3-dehydrocholic acid (3-DHCA), total Bas,unconjugated BAs | gut microbiota-FXR signaling pathway |
| Yan,2024 | China | SCZ | ALL | SCZ(59)  HC(36) | Case control | 95 | LC-MS | clinical diagnosis | plasma | targeted | TG (18:1_36:1), TG (22:4_32:0), TG (22:5_32:1) and CE(16:1) | C2, Cer(d18:1/22:0), Cer (d18:1/24:0), Choline, GABA, lysoPCa C18:0, PC aa C36:0, PC aa C36:2, PC ae C34:0, PC aeC38:2, PC ae C40:1, and SM C24:0 | glycerophospholipid metabolism, butanoate metabolism, sphingolipid metabolism |
| Shi,2024 | China | SCZ | Male | SCZ(96)  HC(96) | Case control | 192 | LC-MS | clinical diagnosis | serum | non-targeted | MG(16:0),  TG(15:0/16:1/16:1), LysoPE(20:3/0:0),  PA(16:0/16:0), PC(16:0/18:1),  PE(16:1/18:0) | Tiglylcarnitine,  DG(18:2/22:5), TG(18:1/18:2/22:2),  TG(18:2/18:3/22:6),  TG(18:2/20:4/20:4),  TG(18:2/20:4/20:5), PA(18:0/22:6),  PC(14:0/14:0), LysoPC(22:6/0:0) | fatty acyl pathway, glyceride pathway, glycerophospholipid pathway, sphingolipid pathway, sterol ester pathway |
| Wang,2024 | China | SCZ | Female | ANS (38)  HC(19) | Case control | 57 | LC-MS | clinical diagnosis | plasma | targeted | Cholesteryl esters (CE) (20:3), Cholic Acid (CA) and Glycocholic Acid (GCA) | γ-Aminobutyric Acid (GABA) | NM |
| Yan,2018 | China | SCZ | ALL | SCZ(20)  HC(29) | Case control | 49 | LC–MS | clinical diagnosis | plasma | non-targeted | CE 16:1,  CE 18:1,  CE 18:3,  CE 20:3,  CE 20:4,  CE 22:6, SM(d14:2/26:2),  TG(16:0/18:1/18:1) | LysoPC 14:0,  LysoPC 18:0, p-PC(P-14:0/20:2),  p-PC(P-16:0/18:2),  p-PC(P-16:0/20:4),  p-PC(P-18:0/20:4) | NM |

SCZ, schizophrenia; HC, healthy controls; FESCZ, first-episode schizophrenia; CSCZ, chronic schizophrenia; NMR, nuclear magnetic resonance; MS, mass spectrometry; GC-MS, gas chromatography-mass spectrometry; LC-MS, liquid chromatography-mass spectrometry; CE-MS, capillary electrophoresis-mass spectrometry; GABA, γ-aminobutyric acid; ADMA, dimethylarginine; PC: Phosphatidylcholine; PE, phosphatidylethanolamine; Lyso-PE, Lyso-phosphatidylethanolamine; ADMA, asymmetric dimethylarginine; NM, not mentioned.
